# Dimensional analysis as a tool for biofilm comparative analysis: bridging the interdisciplinary divides

**DOI:** 10.1128/jb.00188-26

**Published:** 2026-08-07

**Authors:** Chelsea M. Middleton, A-Andrew D. Jones

**Affiliations:** 1 Department of Civil & Environmental Engineering, Duke University 3065, Durham, North Carolina, USA; 2 Thomas Lord Department of Mechanical Engineering & Materials Science, Duke University 3065, Durham, North Carolina, USA; 3 National Institute for Theory and Mathematics in Biology, Chicago, Illinois, USA; University of Southern California, Los Angeles, California, USA

**Keywords:** biofilms, comparative analysis, standard methods, dimensional analysis

## Abstract

Biofilms are the predominant lifestyle for bacteria. Biofilm abundance makes understanding their behavior important for different research domains. These domains can have different information and reporting standards. Information reporting standards have been proposed for some biofilm research methods. Standard methods and consensus standards have also been promulgated. We propose dimensional analysis to encourage abundant information reporting and facilitate comparative analysis. Dimensional analysis utilizes physical system parameters to generate generalizable model data. We review dimensionless quantities that can be applied to biofilm studies. We suggest dimensionless quantities for existing standards, with three examples using dimensional analysis to quantitatively compare literature data.

## INTRODUCTION

Bacteria primarily exist in biofilms, comprising ~80% of bacteria outside of the ocean and 20%–80% of the total bacteria on Earth (1). Bacterial biofilms are agglomerations of bacteria, proteins, sugars, and eDNA that adhere to each other and/or surfaces (2, 3). Biofilm formation occurs in three steps: aggregation and attachment, growth and accumulation, and disaggregation and detachment (4). Biofilms have wide-ranging implications: healthcare, 1.7 million hospital-acquired infections annually (5); environmental management, ~90% of primary production in certain aquatic ecosystems (6, 7); the food and beverage industry (8); pipes and plumbing; and aquatic vessel hulls. Researchers across disciplines conduct research with biofilms often without distinguishing formal frameworks for biofilm and planktonic cell research methods and information standards. This can lead to errant research directions; for example, a meta-analysis of 6,873 antibiotic- and antibacterial-coated vascular grafts found no significant effect of the coatings on preventing infections despite earlier evidence in the biofilm literature that biofilm secretions and cell death would nullify many such coatings (9–13). Interdisciplinary biofilm research can be augmented by searchable, quantifiable, reproducible information standards.

Information standardization is one step toward formalizing analytical frameworks (14). For example, J. Allkja et al. (15) proposed a framework of information standards to assess biofilm formation in microplates. The framework highlights important parameters to report: reactor geometry, volume, temperature, humidity, CO_2_ partial pressure, growth media, nutrient concentrations, agonist concentration, shaking speeds, shaking path and diameter, light exposure, inoculant growth phase, and time. These parameters can have coupled impacts on physiological behavior. For example, reactor geometry and shaking speed change nutrient delivery and shear stress on a biofilm, both of which change a biofilm’s metabolism. pH and osmotic strength should also be reported, as these can induce bacterial cell death and vary based on CO_2_ partial pressure, temperature, and cumulative measurement error.

The framework further divides time into the culturing time of the planktonic form, incubation time, and experimental time (15). These times are correlated but not equivalent to the metabolic state of bacteria (16) and are often established by the experimenter, not the bacteria or system (Table 1). This is partly due to the difficulty in defining standard quantifiable metrics to decide, for example, the survival time to translate a murine chronic wound to a human chronic wound or the time and bacterial cell density to determine if a biofilm infection has been established (17, 18). Planktonic bacteria have physiological time scales and associated biomarkers, lag, log, stationary, and decay phases of growth (16). Physiological markers of time in planktonic phenotypes may have analogs in biofilm phenotypes (19). Biofilm behavior can be described as cooperation, mutualism, or altruism either through proximate or ultimate time scaling (20, 21). These behaviors coupled with mechanical stress, chemical and nutrient stress, and mixed community dynamics change as the biofilm grows in ways that are not easily accounted for *a priori* (22). For example, nutrient stress and cooperation have led to three time scales for biofilm initiation: a nutrient depletion time scale, an autoinducer time scale, and a moving front time scale (23).

**TABLE 1:** Overview of physical quantities, their operational definitions, their dimensions, and values and how they are determined

Symbol {dimensions}	Definition	Determination
** u{L/T} **	Fluid velocity, superficial/Darcy velocity, or speed	Set by experimenter, measured
** r{L} **	Distance from the center of rotation to the biofilm surface	Set by system-specific, measured
** $L_{c}\{L\}$ **	Characteristic length or hydraulic diameter	Set by the system, measured or computed area divided by perimeter
** $v\{L^{2}/T\}$ **	Kinematic viscosity	Material property, literature Dynamic viscosity/density
** $\rho \{M/L^{3}\}$ **	Fluid density	Material property, literature
** μ{M/LT} **	Dynamic viscosity	Material property, literature
** ϕ{−} **	Porosity	Material or system property, measured as volume of empty space/total space
** $S_{v}\{L^{-1}\}$ **	Specific surface area per unit volume of solids	System property, total surface area/total volume
** U{L/T} **	Superficial velocity/Darcy velocity	System property, volumetric flow rate/area for porous materials
** $\sigma_{y}\{MLT^{-2}\}$ **	Yield stress of material	Material property, measured using rheometry or literature
** η{M/LT} **	Coefficient of rigidity of biomass	Material property, measured using rheometry or literature
** $D\{L^{2}/T\}$ **	Mass diffusivity coefficient	Material property, literature or measured using two-chamber devices
** $\mu_{\max}\{1/T\}$ **	Maximum specific growth rate	Biological property, literature or measured highest value during exponential phase of biomass versus time graph
** $q_{\max}\{1/T\}$ **	Maximum specific substrate uptake rate	Biological property, literature or measured substrate consumption during the exponential growth phase
** $S_{bulk}\{M/L^{3}\}$ **	Bulk substrate concentration	Set by experimenter, computed
** l{L} **	Biofilm thickness	Biological result, measured
** $L_{p}\{L\}$ **	Distance available for diffusion	System property, literature or measured diffusion time; sqrt(2Dt)
** $X\{M/L^{3}\}$ **	Biomass density	Biological result, measured
** $Y_{XS}\{M/M\}$ **	Biomass yield coefficient	Biological property, literature or measured; biomass produced/mass of substrate consumed
** $K_{s}\{M/L^{3}\}$ **	Half-saturation coefficient; substrate concentration at which the growth rate is half of $\mu_{\text{max}}$	Biological property, measured or literature
** t{T} **	Characteristic time	Time at which growth curve reaches peak
** $\tau \{M/LT^{2}\}$ **	Wall shear stress	Velocity gradient or friction factor method
** λ{T} **	Relaxation time	Time for material to return to its native state after being deformed

**Fig 1:**
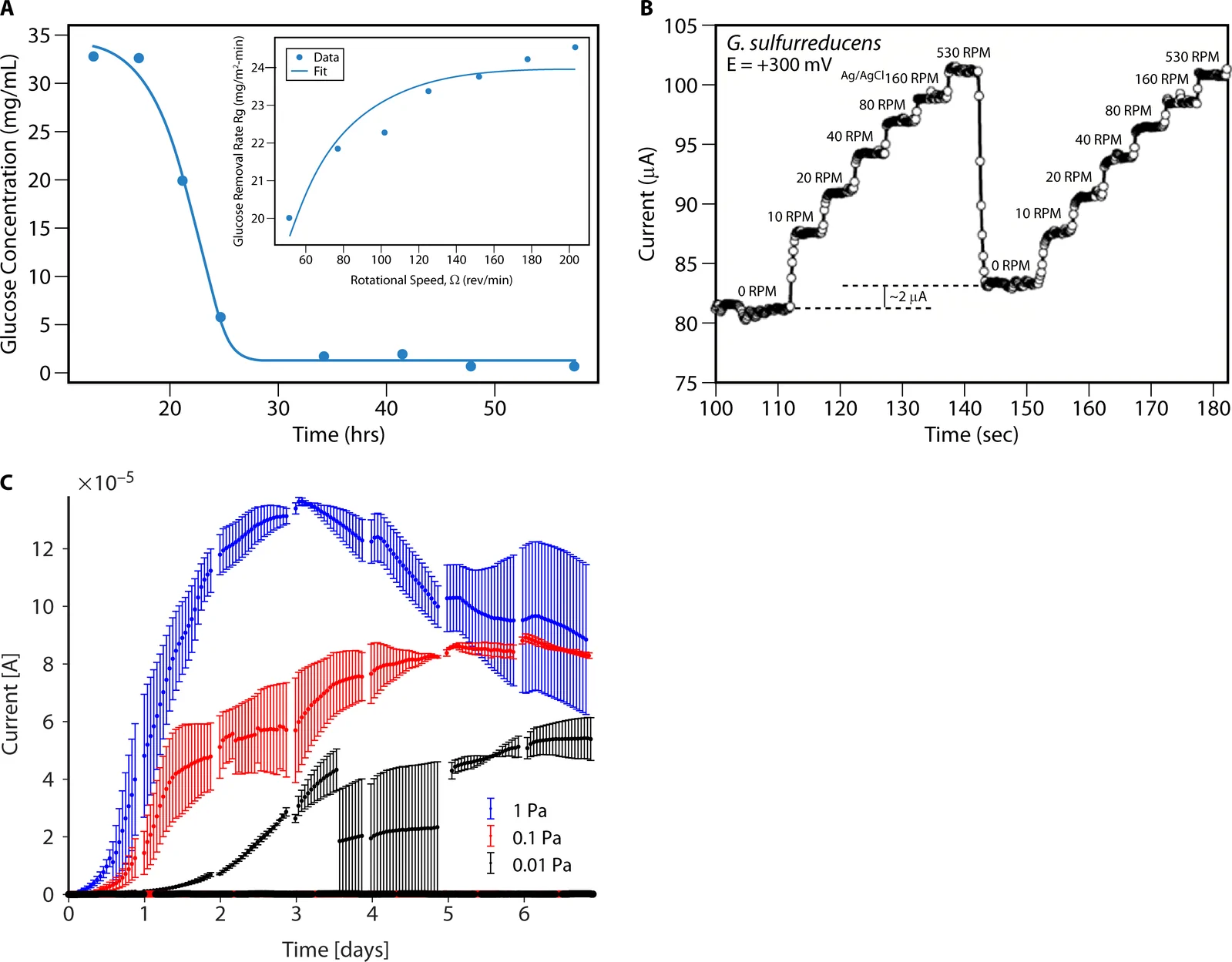
(**A**) Glucose consumption (diffusivity *D* = 6.7 × 10^−6^ cm^2^/s) in a mixed community annular biofilm reactor (inner radius, *r_i_* = 10.5 cm, 0.4 cm outer-inner diameter) ended after the biomass plateau was reached (~72 h); the inset shows repetition at seven rotational rates loaded at 27 mg/m^2^.min (recreated from [22]). (**B**) Current produced by a single species *G. sulfurreducens* with 10 mM acetate (diffusivity *D* = 1.09 × 10^−5^ cm^2^/s) at different rotation rates in a rotating disk (diameter *L*_*c*_ = 0.5 cm) system after 24 h of establishment time (26). (**C**) Current produced by a single species *G. sulfurreducens* with 5.35 × 10^−6^ g COD cm^2^ s^−1^ as acetate over 7 days in a rotating disk system (25) (CCBY 4.0).

The lack of system-, physiological-, or phenotypic-based scaling guidelines can lead to a range of conclusions for the same phenomena. For example, M. Trulear and W. G. Characklis (22) established that increasing shear stress increases substrate utilization for biofilms (Fig. 1) (24). Jones and Buie showed a similar increase in substrate utilization with increasing shear stress from 0 to 3 days in a monoculture *Geobacter sulfurreducens* biofilm. This was the same length of time as that chosen by Trulear and Characklis (Fig. 1B) (25). However, after 3 days, the biofilm subjected to maximum shear that had the maximum metabolic output had a significant decay in metabolic output, while the biofilm subjected to an order of magnitude less shear stress was stable in its output through 7 days (Fig. 1C). Testing for the impact of repeated shear cycling on bacterial biofilm metabolism, Babauta and Beyenal showed shear does not permanently change *G. sulfurreducens* metabolic output (Fig. 1B).

Comparing the phenomena between similar systems with differences in geometry, volume, or nutrient concentrations can complicate analysis of time-based phenomena. For example, Taylor et al. showed a 52-cm packed column mixed community biofilm takes 196 days to reach steady-state permeability reduction while not achieving steady-state substrate penetration (27), while Aufrecht et al. showed a microfluidic sand pack of a two-species biofilm takes 21 h to reach total permeability reduction (28). While species differences may have played an important role in the observed differences in biofilm permeability reduction, these two results cannot be compared without formal quantitative frameworks.

Comparative analysis, comparing results of two or more studies, has always been a part of scientific research. However, there has been a 20-fold increase in formal comparative analysis, like meta-analysis and meta-regression using statistical descriptors, between 2000 and 2019 (29). Meta-analysis requires large data sets to produce accurate and useful results. While the biofilm research community is large, different information reporting across disciplines limits comparative analysis. In addition to groups like the Minimum Information About A Biofilm Experiment (MIABiE) consortia advocating for information standards (30), this paper proposes that increased utilization of dimensional analysis as a quantitative comparative analysis tool will motivate reporting of all quantifiable measurements to maximize the utility of biofilm studies for formal comparative analysis. Dimensional analysis may also improve comparative analysis through mechanistic, in addition to statistical, descriptors (31). This review leverages formal standard methods and several murine protocols for biofilm growth as case studies to show the value and process of dimensional analysis in comparative analysis. Our focus on standards aims for fast uptake while recognizing the high degree of reproducibility and repeatability of these methods in the face of the rising reproducibility crisis (32–36). Our focus on dimensional analysis aims to introduce a more approachable tool for mechanistic discovery than formal mathematical modeling (37–39).

## DIMENSIONAL ANALYSIS IN BIOFILM RESEARCH

Dimensional analysis is a framework for quantitative comparative analysis requiring careful documentation of measurable physical and biological quantities (e.g., standard information criteria described by Allkja et al.) (40, 41). These quantities can include flow rate, nutrient diffusion rate, substrate utilization rate, bacterial growth rate, time, reactor/tissue size, and reactor or tissue geometry. While performing dimensional analysis for biofilms requires careful understanding of biofilm biology and the experimental system’s physics, the process has been formalized as summarized in For those unfamiliar with dimensional analysis. Dimensional analysis both helps identify and depends on accurate identification of the most important inputs for a system. It is a well-suited language for interdisciplinary work so that microbiologists, chemists, and environmental and biomedical engineers can communicate with reduced confusion and facilitate comparative analysis.

### For those unfamiliar with dimensional analysis

Dimensional analysis is common in industrial or engineered systems, like aircraft and chemical reactor design and testing (42). Dimensionless numbers created from this analysis allow scaling and quantitative system comparison from benchtop models to industrial or organism-scaled models. Dimensional analysis is described by the Buckingham Pi Theorem (41, 43). This theorem states that every physical relationship among n physical quantities and measured using k dimensions can be described by i dimensionless numbers $\Pi_{i}$, where i=n-k (41). Physical quantities, n, include known, decided, or documentable parameters like temperature, nutrient concentration, reactor volume, media flow rates, as well as experimentally determined parameters, sometimes from literature, like diffusion constants, substrate utilization rates, and growth rates (Table 1). There are four dimensions that are important to most biofilm studies: mass, length, time, and temperature, represented by M, L, T, and θ when performing dimensional analysis. Others could include current and luminous intensity. Units of common variables, like dynamic viscosity, $\mu \left[=\right]\text{cP}=10^{-3}\text{ Pa.s}$ are represented with the dimensions of pressure times time or force divided by area times time, in terms of the dimensions $M\frac{L}{T^{2}}∙\frac{1}{L^{2}}∙T=\frac{M}{LT}$.

For example, consider a liquid moving through a pipe. If the liquid has a density ρ and dynamic viscosity μ flowing at a velocity u through a pipe of diameter D, there are n=4 physical quantities. Density is measured using mass {M} divided by volume or a length cubed {$L^{3}\}$}. Dynamic viscosity is measured using mass divided by length and time $\left\{\frac{M}{LT}\right\}$. Velocity is measured using length divided by time {L/T}. Diameter is measured using length {L}. There are then k=3 dimensions, mass, length, and time. This results in i=n-k=1 dimensionless number. To find this number, an equation is set up as


$$\Pi_{1}=\rho^{a}\mu^{b}u^{c}D^{d}={\left(\frac{M}{L^{3}}\right)}^{a}{\left(\frac{M}{LT}\right)}^{b}{\left(\frac{L}{T}\right)}^{c}L^{d}$$

For $\Pi_{1}$ to be dimensionless, the exponents of each physical unit must add to 0, or


$$\begin{array}{lr} M: & a+b=0\\ L: & -3a-b+c+d=0\\ T: & -b-c=0 \end{array}$$

There are four unknowns and three equations; hence, one of the unknown exponents must be assigned an arbitrary value, typically 1. One method for deciding which physical quantity to assign an arbitrary exponent value is to match dimensionless numbers with similar physical quantities (Table 2). For example, many of the dimensional numbers in Table 2 are proportional to a length; hence, in this case, we could assign d=1. This results in the dimensionless number $\Pi_{1}=\rho^{1}\mu^{-1}u^{1}D^{1}$, also known as the Reynolds number, equation (1), $\text{Re}=\frac{\rho uD}{\mu }=\frac{uL_{c}}{\nu }=\frac{uL_{c}}{\mu /\rho }$, where kinematic viscosity ν measures force-independent resistive flow {$L^{2}/T\}$}.

**TABLE 2:** Dimensionless groups used in biofilm research or with the potential to be used in biofilm research

Eq. #	Dimensionless group name	Equation	Biological interpretation
(1)	Reynolds	$Re=\frac{uL_{c}}{\nu }$	How flow affects nutrient transport, bacterial adhesion, and detachment rates (44)
(2)	Porous Reynolds	${\text{Re}}_{p}=\frac{\phi U}{\left(1-\phi \right)S_{v}\nu }$	How fluid is flowing through a biofilm-clogged area (25)
(3)	Hedstrom number	$\text{He}=\frac{\rho \sigma_{y}L_{c}^{2}}{\eta^{2}}$	How biofilm will behave in flow, knowing they can behave like elastic solids and liquids (45)
(4)	Sherwood	$Sh=Sc^{n}Re^{m}$	How effectively nutrients can pass through the surrounding bulk fluid, influencing nutrient access (46)
(5)	Schmidt	$Sc=\frac{\nu }{D}$	How efficiently nutrients can diffuse through the fluid boundary layer to reach biofilm (46)
(6)	Peclet number	$\text{Pe}=\frac{uL}{D}$	Determines whether a biofilm is limited by how quickly nutrients diffuse through the boundary layer or by how fast they are delivered by the flow (47)
(7)	Biot	$\text{Bi}=\frac{k_{m}L_{c}}{D}$	Determines whether nutrients and other agents are uniform throughout the biofilm (48)
(8)	Biomass yield	$Y_{XS}=\frac{\mu_{\max}}{q_{max}}$	How efficiently microbes turn nutrients into more biofilm material (38)
(9)	Damköhler (biofilm)	$\text{Da}=\frac{r_{\max}}{\frac{D}{l^{2}}S_{bulk}}$	Determines whether biofilm is limited by how efficiently its microbes consume nutrients or by how quickly nutrients can reach them (42)
(10)	Damköhler (convection)	$Da=\frac{kL_{c}}{u}$	Determines if the system is limited by external mass transport or internal reactions (49)
(11)	Damköhler (diffusion)	$Da=\frac{kL_{c}^{2}}{D}$	Determines if biofilm is an efficient consumer of substrates or a diffusion-limited system (49)
(12)	Theile modulus	$\phi^{2}=\frac{kL_{p}^{2}}{D}$	Determines if the system is limited by diffusion or reaction kinetics (particle surface) (42)
(13)	Theile modulus (biofilm)	$\phi^{2}=\frac{\mu_{\max}XL_{c}^{2}}{Y_{XS}DK_{s}}$	Determines if the system is limited by diffusion or reaction kinetics by defining the depth of the boundary layer (Monod biofilm) (38, 42, 50)
(14)	Fourier number	$\text{Fo}=\frac{t}{L_{c}^{2}/D}$	How efficiently the concentration profile inside the biofilm adjusts to external changes (51)
(15)	Dimensionless shear (inertia)	$\tau^{*}=\frac{\tau }{\frac{1}{2}\rho u^{2}}$	Compares how hard flow is acting on biofilm to how well it’s stuck to a surface (coefficient of friction = surface shear) (25)
(16)	Dimensionless shear (porous inertia)	$\tau^{*}=\frac{10}{\phi^{2}}{\text{Re}}_{p}^{-1}$	Compares fluid’s ability to tear biofilm compared to how well the biofilm is stuck together (porosity/volume ratio) (25)
(17)	Dimensionless shear (viscous)	$\tau^{*}=\frac{\tau L_{c}}{\nu u}$	Compares fluid’s ability to deform biofilm compared to how well the biofilm is stuck together (resistance to flow) (52)
(18)	Deborah number	$\text{De}=\frac{\lambda V}{l}$	How to determine if the structure behaves more like a solid or a liquid under specific conditions (same as Weissenberg in steady flow) (53)

We explicitly link specific dimensionless numbers to standard methods and murine protocols to enable those new to dimensional analysis to find appropriate dimensionless numbers (Table 4). However, there are some instances (Fig. 4) where that is insufficient. One method to derive new, or modified, dimensionless numbers is to leverage a complete equation and use all the parameters therein to form the dimensionless number(s) (39). The equation does not have to be solved or solvable. For example, the Monod equation can lead to the Theile modulus (equation 13) if you also consider nutrient diffusion limits growth of a biofilm. Another method for deriving new or modified dimensionless numbers is through intuition and experience with the biological system. This helps narrow the list of all physical quantities to only the necessary and sufficient. For example, nutrient diffusion is almost always necessary, while protein fold rates can be ignored. Including more physical quantities than necessary, or deciding the “wrong” arbitrary exponent, or arbitrary exponent value are not critical errors. The “wrong” arbitrary constant would result in a dimensionless number raised to a larger or smaller power than common. In the above example, if we had assigned d=2, we would have gotten ${\Pi_{1}=\left(\frac{\rho uD}{\mu }\right)}^{2}=\text{R}{\text{e}}^{\text{2}}$. Similarly, if there are too many physical quantities, then there will be more dimensionless numbers than necessary. These can be combined through multiplication and division to produce new, potentially the “correct” dimensionless number (equation (4), the Sherwood number). Dimensionless numbers are not physical laws; their utility comes through interpreting data or designing experiments with a reduced number of physical quantities in the dimensionless groups that must be varied. For a detailed explanation of the method, see Bridgman 1947 (43), while for an intuitive explanation, see Chapter 5 of Mahajan 2014 (54).

### Dimensionless numbers for biofilm characterizations

There are two dimensionless numbers that have gained traction in biofilm research and four biofilm-specific dimensionless numbers that would benefit from wider usage in biofilm research. The two common dimensionless numbers that have found traction include the Reynolds number and the Sherwood or Peclet number. Biomass yield, the Hedstrom number, the Damköhler number, and the Thiele modulus have been used before in bioprocess engineering, wastewater engineering, and biofilm analysis.

The Reynolds number, Re, equation (1), describes any Newtonian fluid, fluids that behave similarly to water and oil but differently from honey or ketchup, under flow through the ratio of inertial forces to viscous forces. For a biofilm system, it helps measure whether the flow over the film is smooth (laminar) or chaotic (turbulent). Wagner et al. mapped Reynolds number onto changes in the EPS matrix chemistry (44). The Reynolds number can also be used to match systems from one scale (e.g., size or geometry) to another. Jones and Buie matched the porous Reynolds number of a device modeled after an up-flow anaerobic sludge digester or packed bed reactor to the Reynolds number of a rotating disk system (25). The porous Reynolds number, ${\text{Re}}_{p}$, equation (2), is based on the superficial velocity, $U_{0}$; the inverse of the surface area to volume ratio, $S_{v}$; and the measured porosity, ϕ (Table 2) (25). The porous Reynolds number can also be used for flow through soils, sands, and fabrics.

The Hedstrom number, He, equation (3), is used in wastewater engineering research to describe the flow of biomass in pipes. This is necessary because biomass, the mix of bacteria, EPS, and liquid, like mixed liquor suspended solids or a creeping biofilm, is a non-Newtonian fluid and thus cannot be described by a kinematic viscosity, ν; hence, the Reynolds number cannot be used. The Hedstrom number is used to describe the transport of biomass in piping systems, where $L_{c}$ is a pipe inner diameter; $\sigma_{y}$ is the yield stress, the force over area required for the biomass to start moving like a fluid; ρ is the biomass density; and η is the coefficient of rigidity, which is similar to the viscosity of a Newtonian fluid (45).

Chemical mass transport, of species like nutrients, antibodies, oxygen, and carbon dioxide, can be described using either the Sherwood number, Sh, equation (4), or the Biot number, Bi, equation (7), depending on which measurements are available for non-diffusive flux. For non-diffusive flux that can be described using velocity, the Sherwood number is useful. To determine a Sherwood number, the Schmidt number, Sc, equation (5), is needed. The Schmidt number is the ratio of the kinematic viscosity to the diffusivity, D, of the material in question. This number helps determine if diffusion or convection dominates transport phenomena. The product of the Schmidt number and the Reynolds number raised to empirically determined parameters is the Sherwood number. The mass Peclet number, Pe, equation (6), is a special case of the Sherwood number describing the balance of advection of a species to the diffusion of a species when the exponents of the Sc and Re are one (47). The Biot number is a more empirical relationship for more complex situations, like mass transport through tissues or oil and water interfaces. To compute this, the mass transport coefficient, $k_{m}$, is the slope from measurements of concentration differences between the source, $c_{0}$, and a chemical sensor, $c_{2}$, a fixed distance into the system as a function of time, assuming no reactions (55).


$$\log\frac{{c_{0}-c}_{2}}{c_{0}-c_{1}}=k_{m}A(t_{2}-t_{1})$$

Translating results between experimental scale and system scale by matching the Reynolds, Hedstrom, or Sherwood numbers may not be accurate if the mass flux or reaction kinetics are coupled, and additional dimensionless numbers should be used (56). The simplest dimensionless number that describes reaction kinetics is the Damköhler number, equations (9)–(11). For a first-order reaction rate, k, where convective mass transport dominates, the variables are k, $L_{c}$ for characteristic length, and u for velocity. If diffusion dominates the reaction, the focus is on the boundary layer. This involves the same k, $L_{c}$, and the diffusion coefficient D. A biofilm-specific Damköhler number for the production of chemicals, equation (9), (42), uses a substrate with a concentration $S_{bulk}$, biofilm thickness l, and a maximum substrate conversion rate, $r_{max}$.

For catalyzed reactions, the Thiele Modulus, $\phi^{2}$, equations (12)–(13), is more appropriate. The pore length of the catalyst, $L_{p}$, is the characteristic length in the original derivation.

This latter number begins to get closer to an appropriate system for a biofilm, not just a bioreactor. A biofilm-specific Thiele modulus, equation (13), defines the biofilm as a catalyst, with biomass density, X; growth rate, $\mu_{\text{max}}$; substrate yield, $Y_{XS}$; and half-maximum concentration, 𝐾_*s*_ (38, 42).

L. Shuler et al. (42) used the half-maximum concentration 𝐾_*s*_ as the effective concentration, while C. Picioreanu et al. (38) used the concentration in bulk, this distinction should be noted when reporting the Thiele modulus, where the former may not be known *a priori,* as experimental conditions may alter 𝐾_*s*_. Modifications of this Thiele modulus have included the Growth number, *G*, a ratio of maximum biomass growth rate to maximum substrate transport rate,


$$G=Y_{XS}•\phi^{2}\frac{K_{S}}{S_{bulk}}=\frac{\mu_{\text{max}}Xl^{2}}{S_{bulk}D}$$

where the biofilm thickness l is the characteristic length, and an effective reaction rate is the product of the biofilm density, the growth rate $\mu_{max}$ over the concentration of nutrients in the $S_{bulk}$ (38, 57). Another modification of the Thiele modulus accounting for transport and substrate depletion within the biofilm is the δ number (50)


$$\delta^{2}=\frac{{Y_{XS}DS}_{bulk}}{\mu_{\text{max}}Xh^{2}}$$

where h is the boundary layer thickness. This is an inverse analog to the Thiele modulus accounting for the bacteria acting as a catalyst for the reaction of the nutrients, focusing on substrate depletion rather than biofilm growth. Inverted dimensionless groups remain dimensionless.

The mass transfer Fourier number, Fo, equation (14), describes a spreading process through a ratio of species diffusion rate to the rate of species storage. For biofilms, this can be used to describe how efficiently the concentration profile for something like an antibiotic inside the biofilm adjusts to external changes in the concentration (51). It can also be used to describe the spread of bacteria through a medium if those bacteria spread primarily through diffusion, like *S. aureus* or motility-deficient *P. aeruginosa*. A higher number represents a faster rate of diffusion. In this equation, D is the diffusivity, t is the characteristic time, and $L_{c}$ is the length of a body.

The shear stress, τ, {$M/LT^{2}$} is the force acting parallel to a surface used, for example, to quantify mechanical abrasion removal techniques of biofilms. The dimensionless shear, $\tau^{*}$, can be expressed in two different ways, depending on the forces and length scale being considered. For inertia-dominated flows, such as ocean currents and plumbing away from the wall, the dimensionless shear $\tau^{*}$ is dependent on the fluid velocity and the fluid density, equation (15). For a fluid viscosity, μ, dominated phenomena, like blood flow or near-wall flows, the dimensionless shear is given by equation (17). Since dimensionless shear is a scaling of shear, the authors have reported both (58). However, multiplying equation (15) by equation (17) transforms one to the other, with an additional factor of 2.

The Deborah or Weissenberg number, equation (18), describes the behavior of a viscoelastic material under linear stress, generally a steady flow of air or water, as a ratio of the relaxation time of the material to the experimental time. The Deborah number has not been reported in biofilm literature but has been useful for describing the mucosalivery fluids during aerosolization from sneezes and coughs in the nasal cavity or lungs (53). The authors hypothesize that this could be used to describe the relaxation times of biofilms under linear stress. The similarly defined Weissenberg number instead focuses on non-linear rheological stresses (59).

## STANDARD METHODS

Comparative analysis of biofilms could leverage standards that are “tools… to compare different measures” (60). The 2022 Biofilm Research-Industrial Engagement Framework (61) measured the alignment between fundamental scientific understanding and value to industrial translation of biofilm study tools, including standard methods. The framework suggested that animal models, mathematical models, and dental biofilms were well aligned; that wound models were useful for research; and that plate assays were useful for industry, while tissue and microfluidic models were of low value for either. For example, study methods for evaluating the effects of antibiotics on biofilms *in vitro* are not viewed as facile, consistent, or scientifically illuminating enough to justify using them. Many researchers instead rely on planktonic tests (Kirby-Bauer [62] and optical density (OD)-based microtiter assays [63]) as the preliminary data to justify *in vivo* animal models. Dimensional analysis may bridge this alignment mismatch.

The 2022 Biofilm Research-Industrial Engagement Framework (61) identified the 11 ASTM International (ASTM) standard methods, three International Organization for Standardization (ISO) standards, and one American Association of Textile Chemists and Colorists (AATCC) standard for intentional and replicable methods of biofilm growth (Table 3).

**TABLE 3:** Time, volume, species, flow regime, and reactor of ASTM and ISO standards^*a*^^,^^*b*^

Method	Establishing duration	Total time	Species	Volume	Reactor	Transport
ASTM E2799	16–18 h	24 h	*P. aeruginosa*	200 µL	MBEC/shaker	Diffusion
ASTM E2647	30 h	54 h	*P aeruginosa*	Continuous	PFR/drip	Laminar
ASTM E3151	24 h	24 h	*S. epidermidis*	0.5 mL	Tubes/shaker	Diffusion
ASTM E3180 (64)	24 h	48 h	*B. subtilis*	N/A	Plate/static	Diffusion
ASTM E2562	24 h	48 h	*P. aeruginosa*	500 mL	CDC/CSTR	Turbulent
ASTM E2196	–	48 h	*P. aeruginosa*	250 mL	CSTR/rotating	Turbulent
ASTM E3161	24 h	48 h	*P. aeruginosa* or *S. aureus*	325 mL	CDC/CSTR	Turbulent
ASTM E2871	24 h	48 h	*P. aeruginosa or S. aureus*	325 mL	CDC/CSTR	Turbulent
ASTM E3321	20–24 h	96 h	*E. coli*	Continuous	Catheter/AUM	Laminar
ISO 4768	–	48 h	*S. epidermidis*	20 ml	Vessel/static	Diffusion
Abscess model	–	10 days	*P. aeruginosa*		Mouse	Diffusion
Burn wound model	–	11 days	*P. aeruginosa*		Mouse	Diffusion

^*a*^ –, information that is not specifically stated within the listed protocol.

^*b*^ N/A, not applicable.

We omit ISO 11731, as it only recognizes that *Legionella* may come from biofilms but does not grow them, and ISO 20679, which requires biofilm growth on full-scale ships. Additional methods include, for example, the Kirby-Bauer disk diffusion assay for planktonic bacteria and the crystal violet microtiter plate assay for biofilms (33, 65). There are a few consensus-based murine research standards for studying wound and skin biofilms (17, 66). For example, Pletzer et al. promulgated an abscess model using a cystic fibrosis-derived *P. aeruginosa* strain showing higher survival rates and longer clearance than with wild type (17, 67). Brandenburg et al. promulgated a burn wound model on a Sprague-Dawley rat model, similarly showing long survival rates (68). In this section, we identify different parameters like transport type, reactor and reactor size, and bacterial species that may enable scaling and dimensional analysis.

There are two groupings between growth apparatuses in Table 3: either the biofilm is being grown in a continuous flow reactor or in a fixed volume container. Four of these standard methods operate for 48 h. The standards vary in volume from 200 µL to 500 mL, while one operates continuously. The standards explicitly state the times and nutrients established for specific organisms, the predominant organism being *P. aeruginosa,* which makes these standards widely applicable due to the ubiquity of the organism.

**Fig 2:**
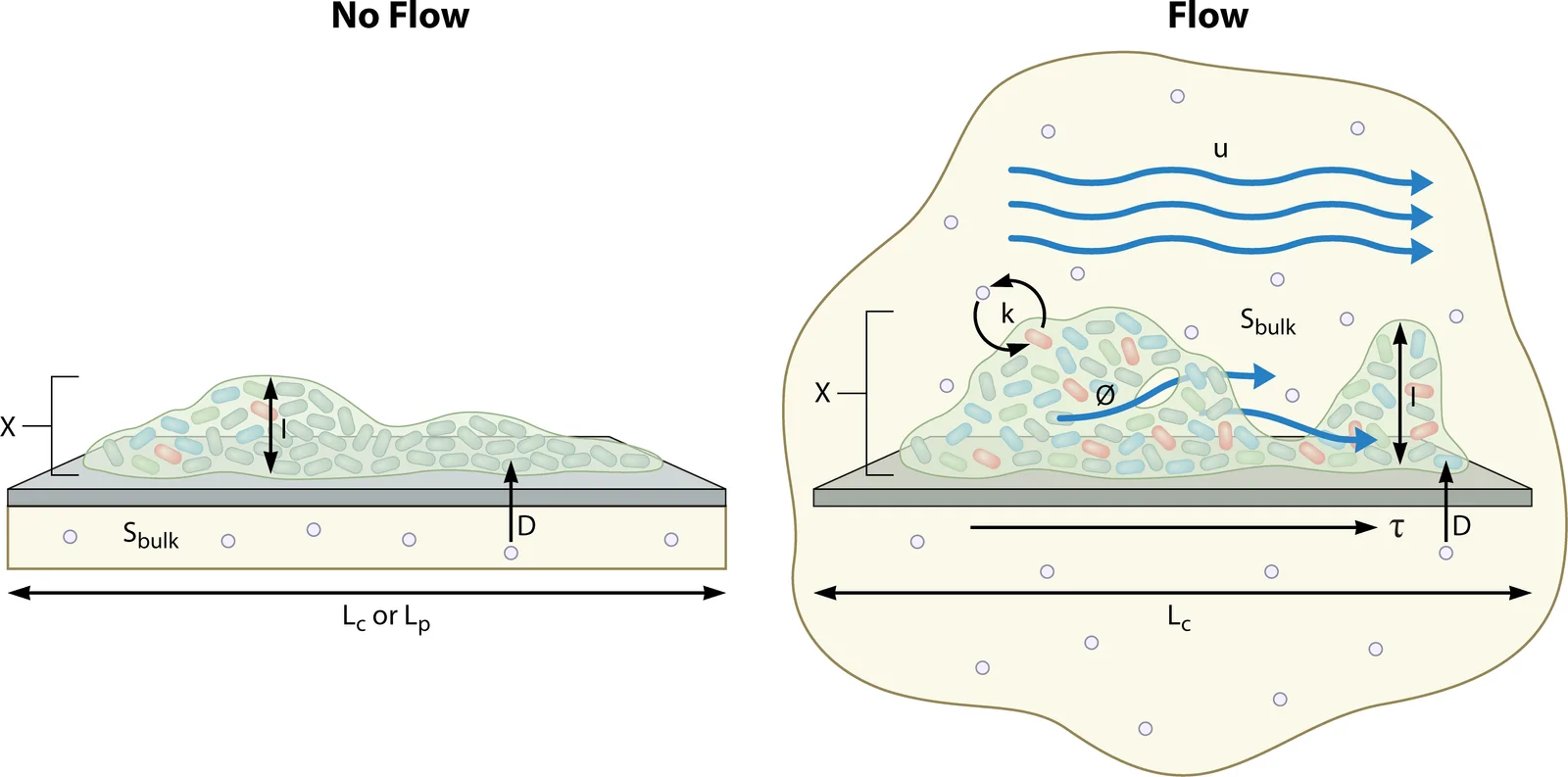
Under no flow conditions, limited nutrient transport leads to slow biofilm development over days to weeks, often resulting in thick but heterogeneous structures. Flow enhances nutrient delivery, adding shear stress, promoting attachment at low rates and detachment at higher rates, and leading to compact biofilms. For shaking incubators, flow and shear can oscillate, and that time scale may need to be included. Additional parameters in the flow case would be the density ρ and dynamic viscosity μ, which can be used to compute the Reynolds and Sherwood numbers. For both cases, growth rate $\mu_{max}$ and biomass yield $Y_{XS}$ would need to be computed from the measured values of substrate consumption and biomass production to compute the Thiele modulus and Damköhler numbers. Temperature, partial pressure of CO_2_, and relative humidity should also be reported.

Reactor types are the distinguishing feature between these methods. Different reactors operate under different conditions mimicking environmental conditions. Low flow conditions are created by little to no velocity/agitation being applied to the system. Under low-flow conditions, limited nutrient transport and low shear stress can lead to slow biofilm development over longer time periods. Moderate flow can be associated with reactors that create laminar flow and lower shear rates, like the drip flow reactor. Moderate flow enhances nutrient delivery while maintaining tolerable shear stress, promoting rapid attachment and stable biofilm growth. High-flow reactors are subjected to very high velocities that are likely to stimulate turbulent flow and high shear on a biofilm. At high flow rates, although nutrient delivery is high, elevated shear stress limits biofilm accumulation through frequent detachment, resulting in thinner biofilms over shorter time scales. This phenomenon is detailed in Fig. 2.

Of the methods that do not involve continuous flow, the apparatuses included are the Minimum Biofilm Eradication Concentration (MBEC) assay, tubes, plates, and tanks. These different geometries hold the nutrients and the culturing cells that could impact growth. ASTM E2799 utilizes the MBEC assay to test disinfectants against biofilms grown on a 96 peg-lid submerged in microtiter plate wells. The biofilm grows on the pegs, where there are fewer nutrients, while planktonic bacteria prefer the broth in the wells (69). Nutrient delivery is limited by diffusion. ASTM E3151 test antimicrobials on biofilms in tubes attached to materials that have tubular geometries. This starts with incubating culture tubes containing tubular materials and biofilm-producing strains of bacteria while shaking. The biofilm adheres to the surfaces of the materials in the tubes, while planktonic bacteria remain in the broth. ASTM E3180 is the colony biofilm model consisting of an agar plate with a sterile semi-permeable membrane placed on agar. The bacteria are inoculated on top of this membrane and statically incubated. ISO 4768 is used to test the properties of the surface of a product by fixing the product to a glass plate and submerging that plate in a vessel holding 20 mL of nutrient medium. This entire system is incubated without shaking. Nutrient delivery in all these methods is diffusion-limited due to low velocities, suggesting that dimensionless numbers are dependent on a diffusion coefficient, like the Biot number, Damköhler (diffusion) number, and the Thiele modulus.

The reactors that experience continuous flow include a drip flow reactor, a catheter, the CDC biofilm reactor, and a continuously stirred tank reactor. The drip flow reactor is a type of plug flow reactor. ASTM E2647 uses this apparatus to model biofilm growth at the air/liquid interface. Water is continuously dripped into the reactor’s influent needle and then dripped out of the effluent line. Inside are coupons where the biofilm is grown that can be extracted from the system. The biofilm is allowed to attach to the coupon first without flow, then the pump is turned on. Because the flow is created by a slow-dripping laminar flow, the shear on the system is relatively low compared to the other laminar flow systems selected. The biofilm can be sampled from the coupon at the end of the test. ASTM E3321 involves using artificial urine as a medium for seeing how *E. coli* biofilms will form in a catheter’s inner tube experiencing continuous flow. The continuously stirred tank reactor in its most basic form is a system that has a feed line, has an effluent line, and is experiencing repetitive mixing. Within the basic growth protocol within the CSTR, biofilms are grown on a coupon. This coupon is attached to the rotating disk element that is creating the mixing aspect in the system. This rotating disk creates a high shear phenomenon within the system. The CDC biofilm reactor is another type of CSTR that helps understand behaviors under high shear. The basic setup is the same. The difference is the setup inside the reactor. The top of the reactor holds eight coupon holders that can hold coupons in triplicate. There is a magnetic stir bar that creates high shear within the reactor. The biofilms are expected to grow on the coupon, while planktonic bacteria will prefer to stay in the nutrient broth coming from the feed line. Continuous flow within this group of methods is generated by a pump circulating media through the system at different flow rates. Some of the reactors, like the different CSTRs, have mixing elements like a stir bar that help add more movement into the system at set speeds. These elements help generate different flow regimes acting on the surface of the biofilms. The flow rates and velocities these systems are typically operated at suggest scaling behaviors that are dependent on velocity, like the Reynolds, Sherwood, and Damköhler (convection) numbers.

Differences in volume are as important as changes in total time and transport regime. One tool relating time, volume, and, to a limited extent, transport, not a dimensionless number, is the hydraulic retention time, HRT =V/Q, where V is the volume and Q is the volumetric flow rate. ASTM E3161 justifies its use of a partially full reactor using the hydraulic retention time. Finally, Table 3 documents the tested bacterial species for each standard method. Growth rates, substrate utilization rates, and half maximum concentration rates are distinct to the species and helpful for identifying if any variation within scaling is due to biological phenomena. Table 4 is a list of the biofilm methods identified in Table 3 with a breakdown of their biological context and a list of relevant physical parameters that will likely be helpful when performing dimensional analysis.

**TABLE 4:** The selected standards with their biological and experimental context and some associated parameters that potentially could be relevant to dimensional analysis

Method	Biological context	Relevant physical parameters	Relevant dimensionless groups
ASTM E2799	*In vitro* method simulates attached biofilms in peg plate to evaluate the efficacy of disinfectants	Time, volume, growth rate, diffusion rate, peg geometry, shear stress (low)	Sc, Sh, Bi,$Y_{XS}$, Da, $\emptyset^{2}$, Fo, $\tau^{*}$
ASTM E2647	Low-shear, continuous flow environmental model for growing and quantifying biofilms on coupons for testing	Time, growth rate, flow rate (1.25 L/day), shear stress, tilt (10°), coupon material, reactor geometry	Re, Sh, Sc, Pe, Bi, $Y_{XS}$, Da,$\emptyset^{2}$, Fo, $\tau^{*}$, De
ASTM E3151	Assesses how effectively materials (tubular) prevent bacterial adhesion and biofilm formation	Time, volume, growth rate diffusion rate, specimen geometry, material, absorbency	Sc, Sh, Bi,$Y_{XS}$, Da, $\emptyset^{2}$, Fo
ASTM E3180	Producing mature biofilms over 8 days on a membrane and evaluating (specifically for taking spore-related antibiotic-resistant measurements)	Time, growth rate, diffusion rate, static, surface geometry, membrane material	Sc, Sh, Bi,$Y_{XS}$, Da, $\emptyset^{2}$, Fo, $\tau^{*}$
ASTM E2562	High shear, continuous flow environmental model for growing and quantifying biofilms on coupons for testing	Time, flow rate, volume, growth rate, rotation speed (125 RPM), shear stress (0.033 Pa), contact angle, coupon material, reactor geometry	Re, He, Sh, Sc, Pe, Bi, $Y_{XS}$, Da,$\emptyset^{2}$, Fo, $\tau^{*}$,
ASTM E2196	Medium shear, continuous flow environmental model for growing and quantifying biofilms on coupons for testing	Time, flow rate, volume, growth rate, rotation speed (200 RPM), shear stress (moderate), coupon material, reactor geometry	Re, Sh, Sc, Pe, Bi, $Y_{XS}$, Da,$\emptyset^{2}$, Fo, $\tau^{*}$
ASTM E3161	High shear, continuous flow environmental model for growing and quantifying biofilms on coupons for testing	Time, flow rate, volume, growth rate, rotation speed (60 RPM), shear stress (0.365 Pa), contact angle, coupon material, reactor geometry	Re, Sh, Sc, Pe, Bi, $Y_{XS}$, Da,$\emptyset^{2}$, Fo, $\tau^{*}$
ASTM E2871	High shear, continuous flow environmental model for growing and quantifying biofilms on coupons for testing	Time, flow rate, volume, growth rate, rotation speed (60 RPM), shear stress (0.365 Pa), contact angle, coupon material, reactor geometry	Re, Sh, Sc, Pe, Bi, $Y_{XS}$, Da,$\emptyset^{2}$, Fo, $\tau^{*}$
ASTM E3321	Evaluates the antimicrobial coatings on urinary catheters (high flow and varying shear) and their ability to resist bacterial adhesion	Time, flow rate, growth rate, shear stress, wall material, reactor geometry	Re, Sh, Sc, Pe, Bi, $Y_{XS}$, Da,$\emptyset^{2}$, Fo, $\tau^{*}$
ISO 4768	Method for evaluating how effectively non-porous materials can prevent the formation of biofilms	Time, diffusion rate, volume, growth rate, coupon material tank geometry	Sh, Bi, $Y_{XS}$, Da,$\emptyset^{2}$, Fo
Abscess model	Method for evaluating infections in internal tissue	Time, diffusion rate, volume, growth rate, clearance rate	Bi, $Y_{XS}$, Da,$\emptyset^{2}$, Fo
Burn wound model	Method for evaluating infections in open tissue	Time, diffusion rate, area, growth rate, wound closure rate	Bi, $Y_{XS}$, Da,$\emptyset^{2}$, Fo

## DIMENSIONAL ANALYSIS WITH STANDARDS AND MODEL SYSTEMS IN PRACTICE

To show the strengths and limitations of dimensional analysis as a form of comparative analysis, we show three examples that leverage both existing dimensionless groups and standard methods. This should serve as a guiding example, not a final analysis of these systems, as the experiments did not report sufficient data for a thorough analysis.

**Fig 3:**
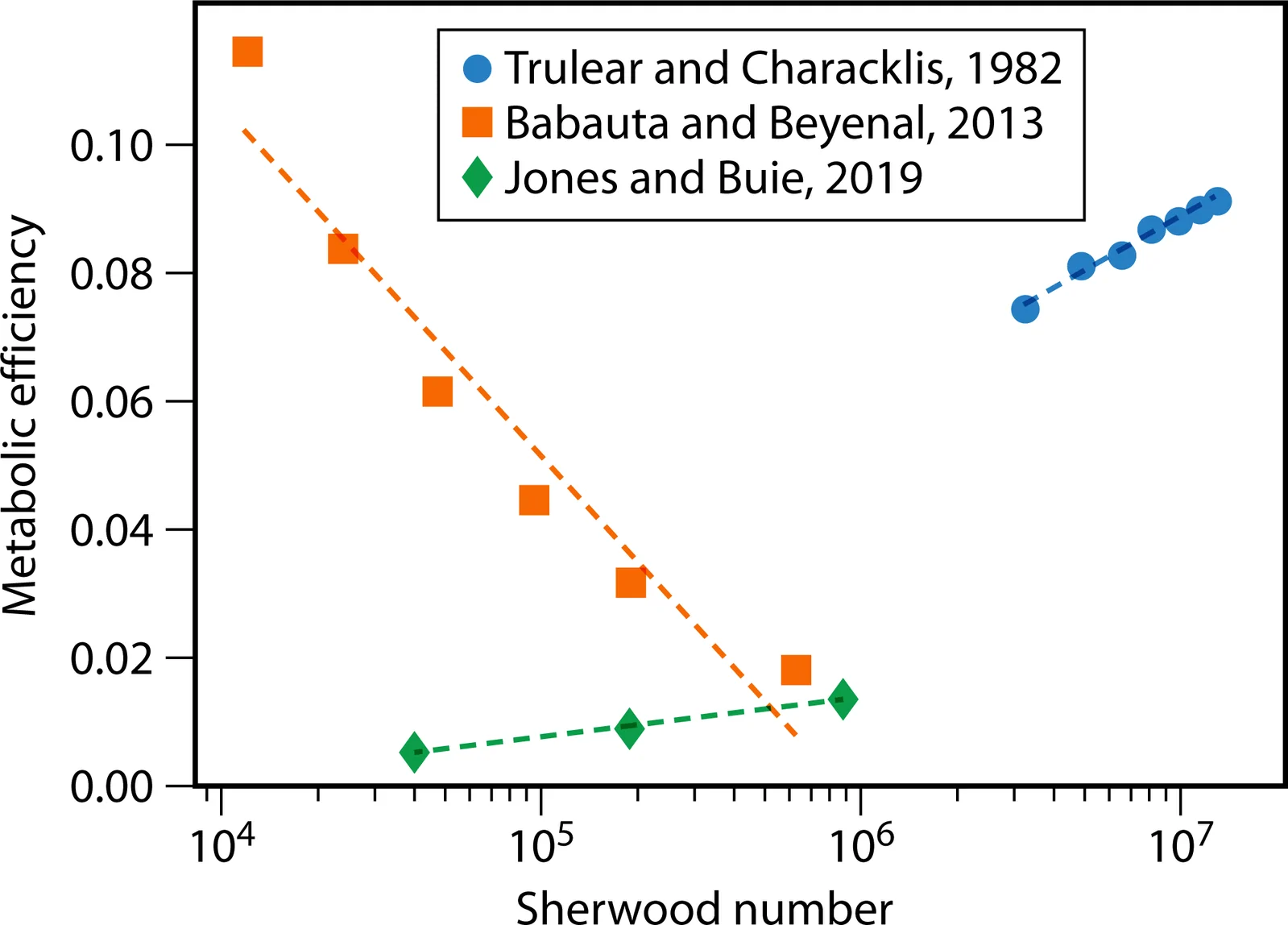
Semi-log plot of the Sherwood number versus metabolic efficiency for the data in Fig. 1. Metabolic efficiency for Trulear and Characklis was found from the inset, dividing the maximum glucose utilization rate by the glucose loading rate. Metabolic efficiencies for Babauta and Beyenal and Jones and Buie were found from the current divided by the theoretical current that could be generated by the acetate flux. Acetate flux is computed from Levich equation (25).

In the first example, the disparate observations in Fig. 1 can be compared using dimensional analysis, as shown in Fig. 3. Here, the metabolic efficiency is plotted versus the log of the Sherwood number (equation 4). All efficiencies, like yields, are dimensionless. Sherwood numbers for the three experiments are calculated using velocities computed from rotational rates, kinematic viscosities were assumed to be close to water, and the diffusion rates were from the literature for glucose and acetate. Metabolic efficiency for Trulear and Characklis was found from the inset of Fig. 1A, dividing the maximum glucose utilization rate by the glucose loading rate. Metabolic efficiency for Babauta and Beyenal and Jones and Buie was found from the current divided by the theoretical current that could be generated by the acetate flux. Acetate flux computed from Levich equation (25). This dimensional analysis shows that there is an impact of initial growth conditions on metabolic efficiency as a function of shear independent of the reactor or species used. Trulear and Characklis and Jones and Buie both established biofilms under shear, while Babauta and Beyenal established the biofilm under static growth conditions. This potentially led to the positive versus negative slopes.

**Fig 4:**
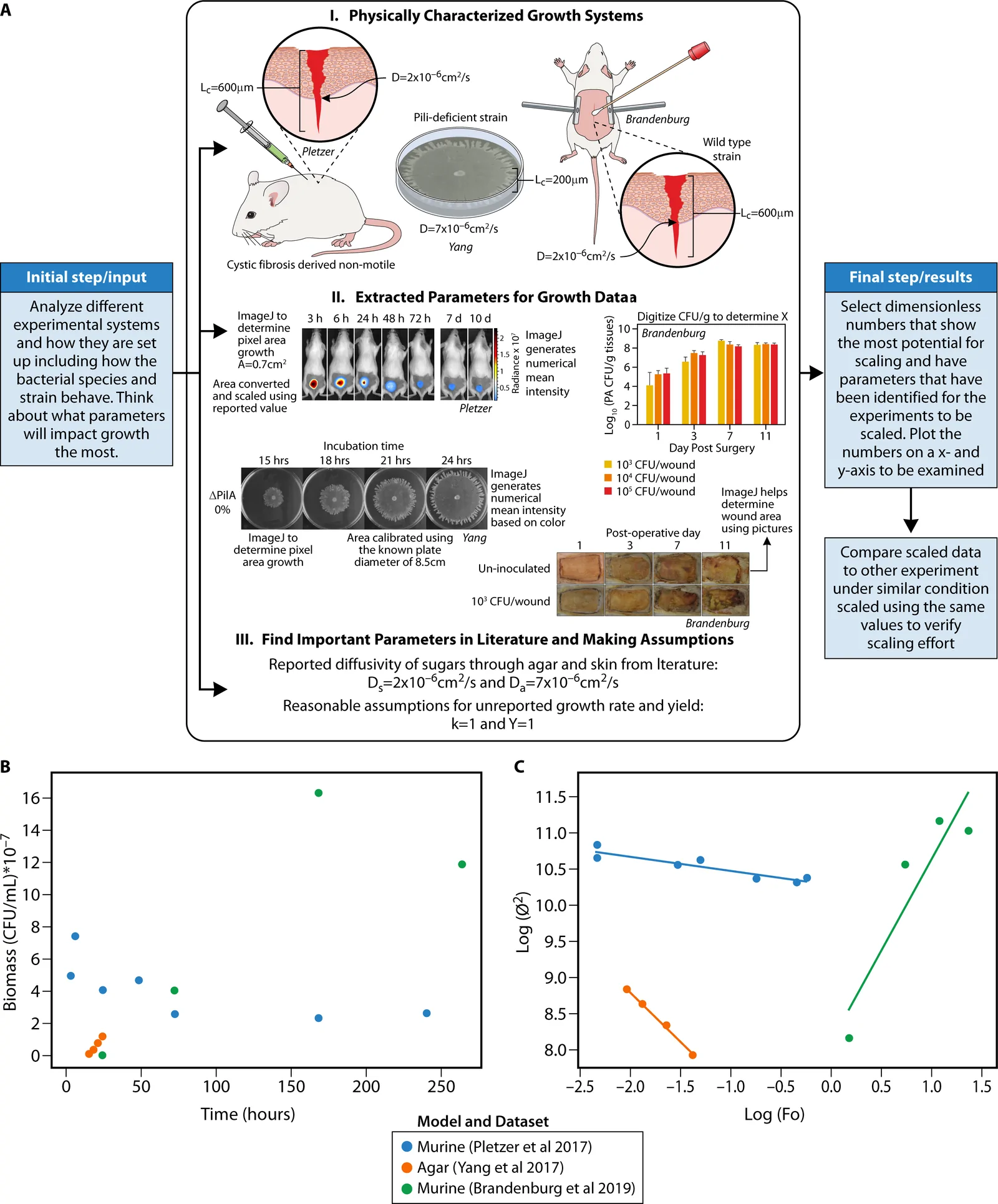
(**A**) Flow chart for dimensional analysis of biofilm spreading. (I) Deciding on appropriate length scales and diffusion of nutrients in the physical system. (II) Extracting area to compute wound closure rates which constrain biofilm spread (17, 68) and biofilm spread rates (70). (III) Identifying parameters from literature, including stating unknowns. (**B**) Biomass versus time and (**C**) log-dimensionally scaled values of biomass growth versus Fourier number.

In the second example, Fig. 4 compares data from two murine models and one agar model of *P. aeruginosa* biofilm growth. These models used different strains of *P. aeruginosa*. Pletzer et al. (17) used a non-motile *P. aeruginosa* derived from a patient living with cystic fibrosis. Yang et al. (70) used a pili-deficient strain. Brandenburg et al. (68) used a wild type. The three systems are physiologically distinct: Pletzer et al. (17) injected bacteria under the skin for a wound abscess murine model; Brandenburg et al. (68) spread bacteria onto a burn wound murine model; and Yang et al. (70) inoculated the center of an agar plate. The Fourier number equation (14) is appropriate to the scale time. For Yang et al.’s agar system, the appropriate time constraint is diffusion of bacteria through agar. The appropriate time constraint on growth in a wound bed is the wound closure rate, expressed as${µ}_{w}$, in cm^2^.s^−1^ computed as opposed to the traditional percent closure per unit time. This is used to create a Fourier number based on wound closure, ${Fo}_{wc}=t{µ}_{w}/L_{c}^{2}$. To find ${µ}_{w}$, a fit line for the wound closure area was found, and a first-order linear approximation of each function was used (Fig. S1a ). To model the bacterial biomass, the Thiele modulus, equation (13), was used. The average reported diffusivities of sugars through agar (71), $D\approx 7\times 10^{-6}\text{c}{\text{m}}^{\text{2}}\text{.}{\text{s}}^{\text{-1}}$, and glucose through murine tissue (72), $D\approx 2\times 10^{-6}\text{c}{\text{m}}^{\text{2}}\text{.}{\text{s}}^{\text{-1}}$, were used. The average thicknesses of a biofilm in skin (600 µm) and on agar (200 µm) were used as the characteristic length since these were not reported. Since the growth rate of different strains in different environments varies significantly and was not reported, a growth rate and yield of 1 was assumed for the Thiele modulus. Dimensionless scaling of all three data sets resulted in linear relationships with $R^{2}$ values between 0.845 and 0.997 (SI Figure A1.b). While the data from these papers were incomplete, for example, biofilm thickness over time was not measured or reported, this approximation initiates interlaboratory, interstudy, and interspecies comparisons. For example, similarities in the sign of the slope of Pletzer et al.’s (17) and Yang et al.’s (70) results confirm the similarities of the cystic fibrosis-derived non-motile *P. aeruginosa* used in the study of Pletzer et al. (17) and the pili-deficient strain used in the study of Yang et al. (70), and those strains’ distinct behavior from the wild type used in the study of Brandenburg et al. (68) (Figure A1.b). This preliminary analysis shows strain-level similarities across distinct platforms, and further work may extend murine model validity to human diseases.

**Fig 5:**
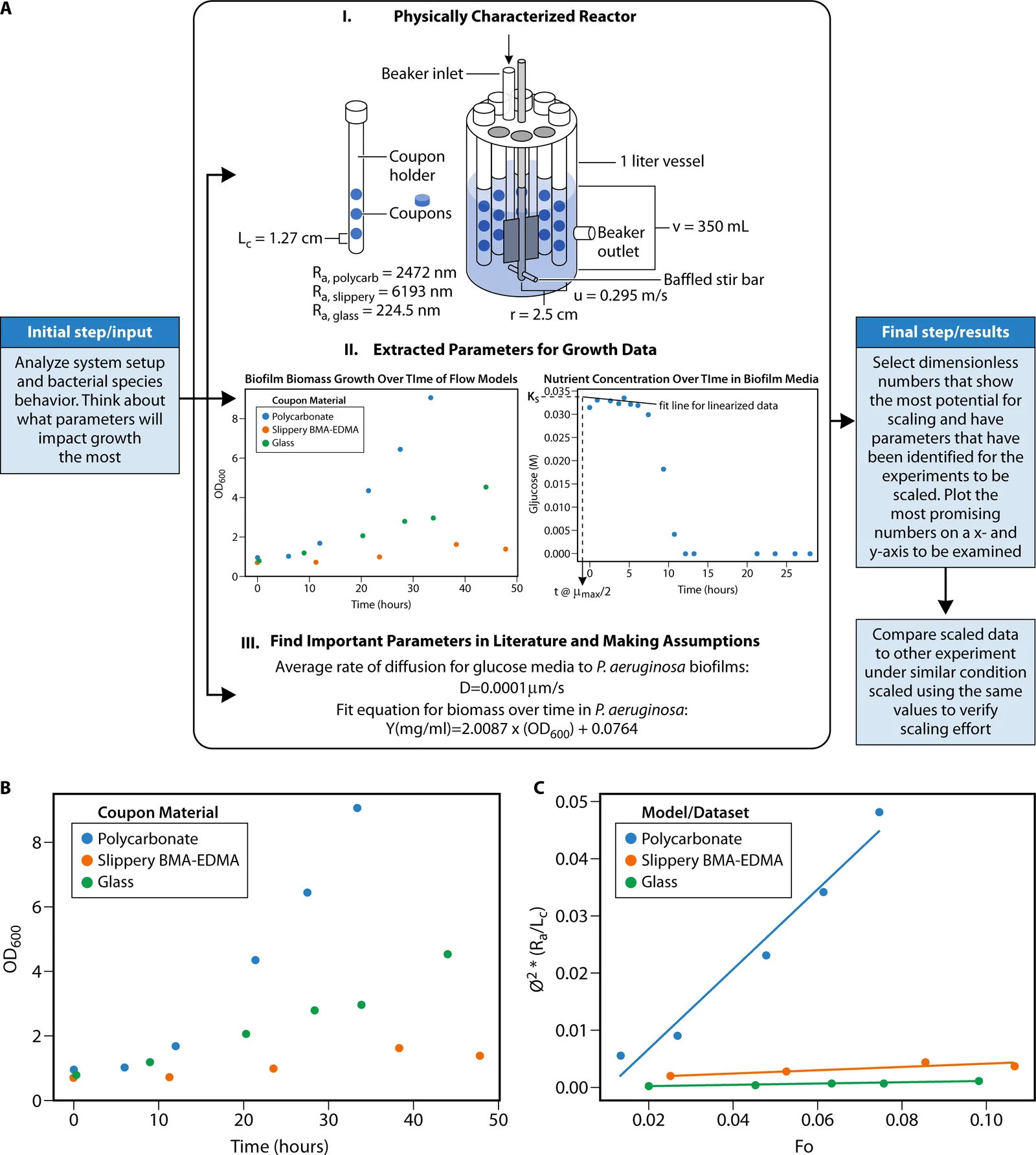
(**A**) Flow chart of dimensional analysis of biomass growth on varying material coupons. (I) Physically characterized reactor; important dimensions are highlighted. (II) Process of extracting parameters for growth data includes original OD and fitting data to find half-maximum concentration $K_{s}$ and maximum-specific growth rate $\mu_{max}$. (III) Literature values used for diffusion and a fit for biomass yield of the existdata. (**B**) Data from the original growth curve of *P. aeruginosa* PA01 biofilm on polycarbonate, glass, and Slippery BMA-EDMA coupons that align to exponential models (66). (**C**) Dimensionally scaled values of biomass growth with an additional roughness correction due to experimental parameters versus Fourier number.

The third example in Fig. 5 compares data from multiple tests run in the previously mentioned CDC biofilm reactor by Valquier-Flynn, Helena et al. (73, 74). The experiments run were all based on ASTM 2562 with the only thing varying between data sets being the coupon material. Because the coupon is the predominant growth surface within the system, changing the material it is made of can have effects on attachment and growth rate. This means it has the potential to scale very differently. These experiments were designed as a way of determining if different polymeric materials have the antimicrobial properties to be effective for the development of medical implants. The trials all used the same *P. aeruginosa* PA01 in TSB under the same velocities, volumes, and on coupons with the same geometry. The differences between the coupons were the surface characteristics of the different materials. The average surface roughness of the specific coupons was measured and reported. Growth data were digitized from the different growth curves. The Fourier number can be used even with its diffusion coefficient because diffusion is always taking place. For this system, an average diffusion rate for TSB nutrients to a *P. aeruginosa* biofilm of $D=3.6\times 10^{-7}\frac{m^{2}}{hr}$ was used; hence, equation (14) is appropriate for scaling. This and using the diameter of the coupon as characteristic length help calculate $Fo=\frac{t}{{(L}_{c}^{2}/D)}$. To model the bacterial biomass, Thiele modulus, equation (13), was used. Known system coupon requirements were used for $L_{c}$. The same diffusion rate was used as in the Fourier number, and $\mu_{max}$ was provided in the literature. Calculations were done for the biomass variable, X, using an equation 2.0087∙OpticalDensity+0.0764 for the linear relationship between glucose utilization and biomass in *P. aeruginosa* was determined by Dong-Ju (75). The average biomass concentration over time was extrapolated from data published by Lee McGill (76). This data set described *P. aeruginosa* biofilm growth in CSP media, which had a primary nutrient of glucose, similarly to the TSB used in the experiment. The rate of change from this relationship was then calculated to determine a reasonable $Y_{XS}$ value. This was used along with data taken from Lee McGill (76) describing glucose utilization of *P. aeruginosa* in CSP media over time to determine a $K_{s}$ variable, which is the glucose measured at $\frac{\mu_{max}}{2}$ based on the Monod equation (SI Figure A2.a). Roughness corrections were applied to the Thiele moduli for each experiment at each time point due to the likelihood that different material roughness would create differences in the surface area available for reactions. This was done using the equation roughness in meters divided by the characteristic length of the coupons provided by Valquier-Flynn et al. (73). The level of detail reported by Valquier-Flynn et al. (73), combined with the nearly identical transport regimes between experiments, allowed finer scaling using the Fourier number and Thiele modulus. This resulted in linear dimensionless scaling relationships, with $R^{2}$ values between 0.803 and 0.968 (SI Figure A2.b). The similarities in slope and magnitude between these scaled lines prove experimental comparability. More surface measurements would likely lead to even finer scaling considering experimental correlations observed between surface skewness and kurtosis and growth rate (67).

These three examples show a range of techniques, limitations, and successes of dimensional analysis as a tool for comparative analysis. In the shear stress example, the Sherwood number was used as described, while metabolic efficiency was computed. In the murine example, a modified Fourier number was derived based on wound closure rates instead of using the existing form. In the reactor model, a modified Thiele modulus was used based on surface roughness. Furthermore, it was assumed which nutrient in agar and tissue was limiting. As more data are collected and reported, quorum sensing, oxygen demand, byproduct, or biomass may be deemed more important. All three examples revealed independent testable hypotheses of finite parameter spaces: metabolic efficiency as a function of shear is a function of the initial shear during biofilm establishment; spread of biofilm is only a function of species motility independent of substrate structure; and biofilm growth rate in high shear stress fluid flow conditions is a function of topographical measurements of the biofilm growth surface. Comparing the conclusions of the examples in Fig. 3 and 4, it might also be worth testing whether biofilm propagation under no flow conditions is species dependent while biofilm propagation under flow conditions is species independent.

## CONCLUSION

Comparative analysis is a critical component of scientific research. However, it requires information and experimental standards to allow for meaningful comparisons. Due to the interdisciplinary nature of biofilm research, recent years have established and called for new reporting standards. We believe that dimensional analysis could serve as a motivating factor for increased reporting. We documented 18 dimensionless numbers that could be used in biofilm research. We identified dimensionless numbers that can be used to analyze results from ASTM, ISO, and consensus standards. We showed three examples of comparative analysis using dimensional analysis, confirming the biological origins of the reported phenomena across multiple research papers that could also lead to new research directions. We hope that recognition of the value and awareness of dimensional analysis will inspire more and better reporting of all experimental parameters.

## SUPPLEMENTAL MATERIAL

Supplemental figures jb.00188-26-s0001.docx Data, models, and statistics of fits used for computing the parameters used in the dimensional analysis.

ASM does not own the copyrights to Supplemental Material that may be linked to, or accessed through, an article. The authors have granted ASM a non-exclusive, world-wide license to publish the Supplemental Material files. Please contact the corresponding author directly for reuse.
