# Supplementary material for: Dimensional analysis as a tool for biofilm comparative analysis: bridging the interdisciplinary divides
Source: J Bacteriol. 2026 Aug 7;208(9):e00188-26. doi: 10.1128/jb.00188-26 (PMC13584748; doi:10.1128/jb.00188-26)
Supplement: Supplementary file 1 [file jb.00188-26-s0001.docx]

**Supplementary Information for “**Dimensional Analysis as a Tool for Biofilm Comparative Analysis: Bridging the Interdisciplinary Divides”

Chelsea M. Middleton^1^ and A-Andrew D. Jones, III^1,2,3*^

1. Department of Civil & Environmental Engineering, Duke University, Durham, NC
2. Thomas Lord Department of Mechanical Engineering & Materials Science, Duke University, Durham, NC
3. National Institute for Theory and Mathematics in Biology, Chicago, IL

*Corresponding Author: andrew.jones3@duke.edu

Data, models and statistics of fits used for computing the parameters used in the dimensional analysis of examples two (Figure A1) and three (Figure A2).

a.

b.

Figure A1. a. Wound closure rate of Pletzer et al.(1) and Brandenburg et al.(2) and for completeness, the rate of spread of P. aeruginosa on agar. b. Log - Dimensionally scaled values of biomass growth versus Fourier number with fit equations and the proportions of variation.

b.

a.

Figure A2. a. Lee McGill (3) describing glucose utilization of *P. aeruginosa* in CSP media over time used to determine a K_s_ variable which is the glucose measured at μ_max/2_ based off the Monod equation. b. Dimensionally scaled values of biomass growth with an additional roughness correction due to experimental parameters versus Fourier number with fit equations and the proportions of variation.

1.	Pletzer D, Mansour SC, Wuerth K, Rahanjam N, Hancock RE. 2017. New Mouse Model for Chronic Infections by Gram-Negative Bacteria Enabling the Study of Anti-Infective Efficacy and Host-Microbe Interactions. mBio 8.

2.	Brandenburg KS, Weaver AJ, Jr., Qian L, You T, Chen P, Karna SLR, Fourcaudot AB, Sebastian EA, Abercrombie JJ, Pineda U, Hong J, Wienandt NA, Leung KP. 2019. Development of Pseudomonas aeruginosa Biofilms in Partial-Thickness Burn Wounds Using a Sprague-Dawley Rat Model. J Burn Care Res 40:44–57.

3.	McGill SL, Yung Y, Hunt KA, Henson MA, Hanley L, Carlson RP. 2021. Pseudomonas aeruginosa reverse diauxie is a multidimensional, optimized, resource utilization strategy. Scientific Reports 11:1457.
